# Maternal vitamin D–related metabolome and offspring risk of asthma outcomes

**DOI:** 10.1016/j.jaci.2023.06.030

**Published:** 2023-08-08

**Authors:** Min Kim, Nicklas Brustad, Mina Ali, Gözde Gürdeniz, Morten Arendt, Augusto A. Litonjua, Craig E. Wheelock, Rachel S. Kelly, Yulu Chen, Nicole Prince, Feng Guo, Xiaobo Zhou, Jakob Stokholm, Klaus Bønnelykke, Scott T. Weiss, Hans Bisgaard, Jessica Lasky-Su, Bo Chawes

**Affiliations:** a Copenhagen Prospective Studies on Asthma in Childhood (COPSAC), Herlev and Gentofte Hospital, University of Copenhagen, Copenhagen;; b Faculty of Health and Biomedical Science, University of Surrey, Guildford;; c the Section of Microbiology and Fermentation, Department of Food Science, University of Copenhagen, Copenhagen;; d the Division of Pediatric Pulmonary Medicine, Golisano Children’s Hospital, University of Rochester Medical Center, Rochester;; e Unit of Integrative Metabolomics, Institute of Environmental Medicine, Karolinska Institutet, Stockholm;; f the Department of Respiratory Medicine and Allergy, Karolinska University Hospital, Stockholm;; g the Gunma University Initiative for Advanced Research (GIAR), Gunma University, Maebashi;; h the Channing Division of Network Medicine, Brigham and Women’s Hospital and Harvard Medical School, Boston;; i the Jiangsu Key Laboratory of Immunity and Metabolism, Department of Pathogenic Biology and Immunology, Xuzhou Medical University, Xuzhou, Jiangsu.

**Keywords:** Vitamin D, metabolomics, pregnancy, childhood asthma, sphingomyelin

## Abstract

**Background:**

Gestational vitamin D deficiency is implicated in development of respiratory diseases in offspring, but the mechanism underlying this relationship is unknown.

**Objective:**

We sought to study the link between gestational vitamin D exposure and childhood asthma phenotypes using maternal blood metabolomics profiling.

**Methods:**

Untargeted blood metabolic profiles were acquired using liquid chromatography-mass spectrometry at 1 week postpartum from 672 women in the Copenhagen Prospective Studies on Asthma in Childhood_2010_ (COPSAC_2010_) mother-child cohort and at pregnancy weeks 32 to 38 from 779 women in the Vitamin D Antenatal Asthma Reduction Trial (VDAART) mother-child cohort. In COPSAC_2010_, we employed multivariate models and pathway enrichment analysis to identify metabolites and pathways associated with gestational vitamin D blood levels and investigated their relationship with development of asthma phenotypes in early childhood. The findings were validated in VDAART and in cellular models.

**Results:**

In COPSAC_2010_, higher vitamin D blood levels at 1 week postpartum were associated with distinct maternal metabolome perturbations with significant enrichment of the sphingomyelin pathway (*P* < .01). This vitamin D–related maternal metabolic profile at 1 week postpartum containing 46 metabolites was associated with decreased risk of recurrent wheeze (hazard ratio [HR] = 0.92 [95% CI 0.86–0.98], *P* = .01) and wheeze exacerbations (HR = 0.90 [95% CI 0.84–0.97], *P* = .01) at ages 0 to 3 years. The same metabolic profile was similarly associated with decreased risk of asthma/wheeze at ages 0 to 3 in VDAART (odds ratio = 0.92 [95% CI 0.85–0.99], *P* = .04). Human bronchial epithelial cells treated with high-dose vitamin D_3_ showed an increased cytoplasmic sphingolipid level (*P* < .01).

**Conclusions:**

This exploratory metabolomics study in 2 independent birth cohorts demonstrates that the beneficial effect of higher gestational vitamin D exposure on offspring respiratory health is characterized by specific maternal metabolic alterations during pregnancy, which involves the sphingomyelin pathway.

## GRAPHICAL ABSTRACT

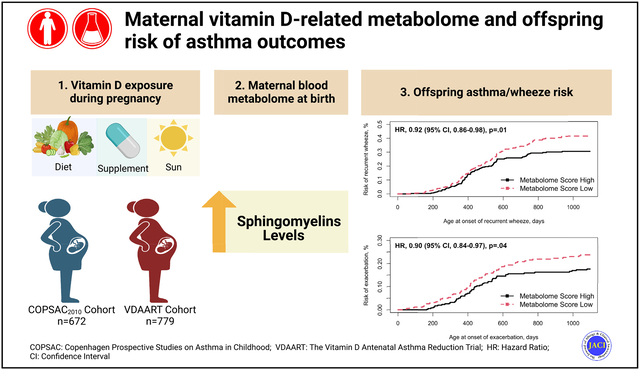

Vitamin D deficiency in childhood has been associated with various adverse health consequences as 25-hydroxyvitamin D (25(OH)D) possesses a range of immune regulatory properties with potential implications for proneness to respiratory infection, allergies, and asthma.^1,2^ Studies have further shown that 25(OH)D deficiency during pregnancy may affect fetal immune programming, consequently increasing the risk of asthma in early childhood.^3,4^ Therefore, we recently performed an intervention study in the Copenhagen Prospective Studies on Asthma in Childhood 2010 (COPSAC_2010_) cohort,^5^ which showed beneficial effects of high-dose versus standard-dose of vitamin D supplementation (2800 IU/day vs 400 IU/day) during pregnancy on episodes of troublesome lung symptoms (TROLS),^6^ persistent wheeze,^7^ and croup.^8^ However, the underlying mechanisms of the protective effects of vitamin D remain elusive, and we hypothesized that they could be disentangled by applying metabolomics profiling during pregnancy.

Metabolomics is the study of endogenous and exogenous metabolites in biological systems, which can be used for uncovering perturbed metabolic pathways due to phenotype differences and for detecting potential new targets for therapeutic intervention. This omics approach has previously been employed to study the response to high-dose 25(OH)D supplementation in critical illness,^9,10^ but only one study of 30 pregnant, Black adolescents has investigated the association between 25(OH)D levels during pregnancy and the maternal metabolome.^11^ The latter study observed 25(OH)D-related metabolic perturbations, but did not investigate the role of this on offspring health outcomes.^11^

Here, we performed untargeted, global metabolic profiling of maternal blood samples from the COPSAC_2010_ mother-child cohort^5,6,12^ aiming to identify metabolites and metabolic pathways linked to 25(OH)D exposure during pregnancy and investigate their association with childhood respiratory health outcomes, which we and others previously found to be affected by gestational vitamin D exposure.^6,7^ Further, the analyses were stratified by the 17q21 genotype, as we previously showed an effect of interaction between this genotype and high-dose vitamin D supplementation in pregnancy on risk of offspring childhood asthma development.^13^ Finally, we used the Vitamin D Antenatal Asthma Reduction Trial (VDAART) mother-child cohort to investigate whether the metabolites linked to 25(OH)D exposure in COPSAC_2010_ were also associated with childhood respiratory health outcomes in an independent cohort as a validation step.

## METHODS

### Study populations

The COPSAC_2010_ cohort study is a mother-child cohort with 736 participating families.^5^ A subset of pregnant women (n = 623) was enrolled in a double-blinded randomized controlled trial (RCT) at pregnancy week 24, where they were randomized 1:1 to a daily dose of 2400 IU/day of vitamin D_3_ supplementation or a matching placebo tablet (Camette A/S, Syddanmark, Denmark) until 1 week postpartum (ClinicalTrials.gov identifier NCT00856947).^6^ Additionally, all women were given 400 IU/day of vitamin D_3_ supplementation during pregnancy as recommended by the Danish National Board of Health. Hence, the study was a dose comparison of 2800 IU/day (high-dose group, n = 315) versus 400 IU/day (standard-dose group, n = 308).

At 1 week postpartum, blood samples were collected from the mothers to measure 25(OH)D level as previously detailed.^14,15^ In addition, all women participated in a concomitant factorial designed, double-blinded RCT of 2.4 g/day of n-3 polyunsaturated fatty acids (PUFAs) during pregnancy (ClinicalTrials.gov identifier NCT00798226). More details of the RCTs can be found elsewhere.^5,6,16^

The VDAART study^17^ is an RCT with parallel design that was conducted in 3 centers across the United States. In this study, 876 pregnant women at high risk of having children with asthma were randomly assigned at 10 to 18 weeks of gestation to high-dose vitamin D (4400 IU/day) or placebo plus a prenatal vitamin containing 400 IU/day vitamin D. Blood samples were collected from mothers at 32 to 38 weeks’ gestation.

Plasma samples for measurement of 25(OH)D level were additionally collected from children at age 6 months in COPSAC_2010_.

### Data collection

#### Clinical outcome.

The COPSAC_2010_ children were followed prospectively for development of asthma, which was diagnosed exclusively by the COPSAC pediatricians, as detailed previously.^5^ In brief, the diagnosis was based on recurrent wheeze (minimum 5 episodes of TROLS lasting at least 3 consecutive days within the preceding 6 months), typical asthmatic symptoms, and rescue use of inhaled short-acting bronchodilator. Children meeting those criteria were prescribed a 3-month trial of inhaled corticosteroids (ICSs), which were thereafter paused to investigate signs of relapse.^16^ Children responding to ICSs and relapsing at cessation were diagnosed with asthma, which is termed persistent wheeze before age 3 and asthma thereafter. Children meeting the criteria for initiation of ICSs as described above regardless of response to the treatment were diagnosed with recurrent wheeze.

From birth to age 3 years, the parents completed a daily diary, recording the occurrence of TROLS and common infections (tonsillitis, cold, acute otitis media, croup, lower respiratory infection). All these variables were reviewed and verified by the COPSAC pediatricians at both scheduled and acute care visits.

Lung function measurements were performed at the 6-year follow-up visit. FEV_1_ and specific airway resistance were measured by a MasterScope Pneumoscreen spirometer (Erich Jäeger, Würzburg, Germany) and MasterScope bodybox (Erich Jäeger), respectively. All measurements were calibrated for age, sex, and height.^18^

At age 6, allergic sensitization was diagnosed from a positive skin prick test (SPT) and/or elevated specific IgE (>0.35 kU/L) against a panel of common food and aeroallergens.^19^

In this study, our primary clinical end points were risk of developing recurrent/persistent wheeze and exacerbations until age 3, number of TROLS episodes until age 3, and lung function and sensitization at age 6. Secondary clinical end points included number of episodes of infections (tonsillitis, cold, acute otitis, croup, lower respiratory infection) until age 3.

For VDAART, asthma/wheeze status and number of episodes of infection were available until age 3 years. During the 3-year follow-up, allergic sensitization was determined by measuring specific IgE against a panel of common food allergens and aeroallergens. At the 6-year follow-up, impulse oscillometry was used to measure resistance at 5 Hz (R5) and at 20 Hz (R20), and spirometry was performed to measure FEV_1_.

#### Metabolomics.

Plasma metabolic profiling on the blood samples from COPSAC_2010_ (1 week postpartum for mothers and 6 months for children) and VDAART (gestation weeks 32 to 38) was carried out by Metabolon, Inc. (Durham, NC), using their HD4 platform. A detailed description of the metabolomics protocol is provided in Metabolomic data acquisition in this article’s Online Repository at www.jacionline.org and elsewhere.^20^

#### 17q21 Genotype.

Participating mothers in COPSAC_2010_ were genotyped for the 17q21 rs12936231 single nucleotide polymorphism with the Illumina Infinium HumanOmniExpressExome BeadChip (San Diego, Calif).

A time line illustrating participant recruitment and data collection can be found in Fig E1 (in the Online Repository at www.jacionline.org).

### Data analysis

Of 738 pregnant women enrolled in the COPSAC_2010_ cohort, 672 had plasma metabolic profiles available at 1 week postpartum (standard-dose vitamin D, n = 276; high-dose vitamin D, n = 286; not enrolled into vitamin D RCT, n = 110). After data quality control processing (see Data processing pipeline in the Online Repository at www.jacionline.org), the datasets consisted of 753 annotated metabolites involved in 9 different metabolic super-pathways. Before statistical analyses, all metabolite levels were log-transformed to normalize the data and z-scored to have mean of 0 and SD of 1.

For VDAART, 779 pregnant women had plasma metabolic profiles measured at weeks 32 to 38. In COPSAC_2010_, metabolic profiles in 565 children at age 6 months were also acquired using the same platform.

#### Maternal vitamin D exposure versus metabolome.

We first investigated changes in the blood metabolome by high-dose versus standard-dose supplementation and by gestational 25(OH)D level by employing univariate and multivariate models using COPSAC_2010_ as discovery cohort. Before analysis, 25(OH)D levels were calibrated for the PUFA intervention, maternal smoking during the third trimester, offspring sex, and season of birth.

Multivariate data analysis was performed by using orthogonal partial least squares (OPLS) discriminant analysis for high-dose versus standard-dose intervention groups and OPLS Y for calibrated 25(OH)D levels. From each model, metabolites with variable influence on projection (VIP) scores toward predictive components higher than 2 were defined as key metabolites reflecting gestational vitamin D exposure and underwent further OPLS and unsupervised principal component analysis (PCA) models. All OPLS models were double cross-validated (cross-validation, n = 10; permutation, n = 1000).

Univariate data analysis was carried out using regression models to investigate how 25(OH)D influenced levels of each blood metabolite. Logistic (binary) and linear regression models were employed for high-dose versus standard-dose and calibrated 25(OH)D, respectively. In logistic models, PUFA intervention, maternal smoking during the third trimester, offspring sex, and birth season were adjusted for. Analyses were adjusted for multiple testing using false discovery rate (FDR).

#### Vitamin D-related metabolome versus offspring clinical end points.

For the second part of this study, vitamin D exposure–related maternal metabolites with VIP scores >2 were investigated for association with offspring clinical end points. Cox regression survival analysis was performed for age at onset of recurrent wheeze, persistent wheeze/asthma, and exacerbations at ages 0 to 3. Quasi-Poisson regression was applied to estimate the effect of key metabolites with an incidence rate ratio (IRR) for common infections and TROLS until age 3. Linear regression models were used for analyzing lung function measurements, while logistic regression was employed for allergic sensitization at age 6. In all models, PUFA intervention, maternal smoking during the third trimester, offspring breast-feeding days, sex, day care start age, and birth season were adjusted for.

#### Validation in VDAART.

We investigated whether the vitamin D exposure–related maternal metabolites from COPSAC_2010_ showed the same associations with clinical end points in VDAART as a validation step.

#### Child age 6 months 25(OH)D and metabolome in COPSAC_2010_.

In addition to maternal metabolome, we acquired 25(OH)D level and blood metabolome in children at age 6 months. First, we repeated the association between vitamin D exposure–related maternal metabolome and offspring clinical end points adjusting for child age 6 months 25(OH)D level. Then, we investigated the association between the vitamin D exposure–related metabolites found in mothers and child clinical end points using the offspring metabolome at 6 months as exposure.

#### Vitamin D treatment in human bronchial epithelial cells.

We investigated the functional effect of vitamin D treatment on the sphingolipid metabolism in human bronchial epithelial cells with or without overexpression of the *ORMDL3* gene located in the 17q21 region.^13^ Herein, we have collected data generated in human bronchial epithelial cell lines (16HBE) treated with 1*α*,25-vitamin D_3_ at 4 concentrations (0 nM, 0.1 nM, 1 nM, and 10 nM, n >10 repeats for each treatment) for 10 hours and analyzed the effect of increasing 25(OH)D_3_ stimulations on measured levels of cytoplasmic sphingolipid product, ie, sphinosine-1-phosphate, by ELISA. More details can be found in Vitamin D treatment of human bronchial epithelial cell lines in the Online Repository at www.jacionline.org.

All statistical analyses were performed in R Studio version 2021.09.2.

## RESULTS

### Baseline characteristics

Baseline characteristics and clinical outcome measures of the mothers and their children as well as differences between the high-dose and standard-dose vitamin D groups for both cohorts are outlined in Table I. Briefly, the only significant difference between the high-dose and standard-dose groups for both VDAART and COPSAC_2010_ cohorts was for 25(OH)D level at 1 week postpartum in COPSAC_2010_ and gestation weeks 32 to 38 for VDAART (both *P* < .0001).

### Vitamin D supplementation and maternal metabolic profile at 1 week postpartum in COPSAC_2010_

**FIG 1.**
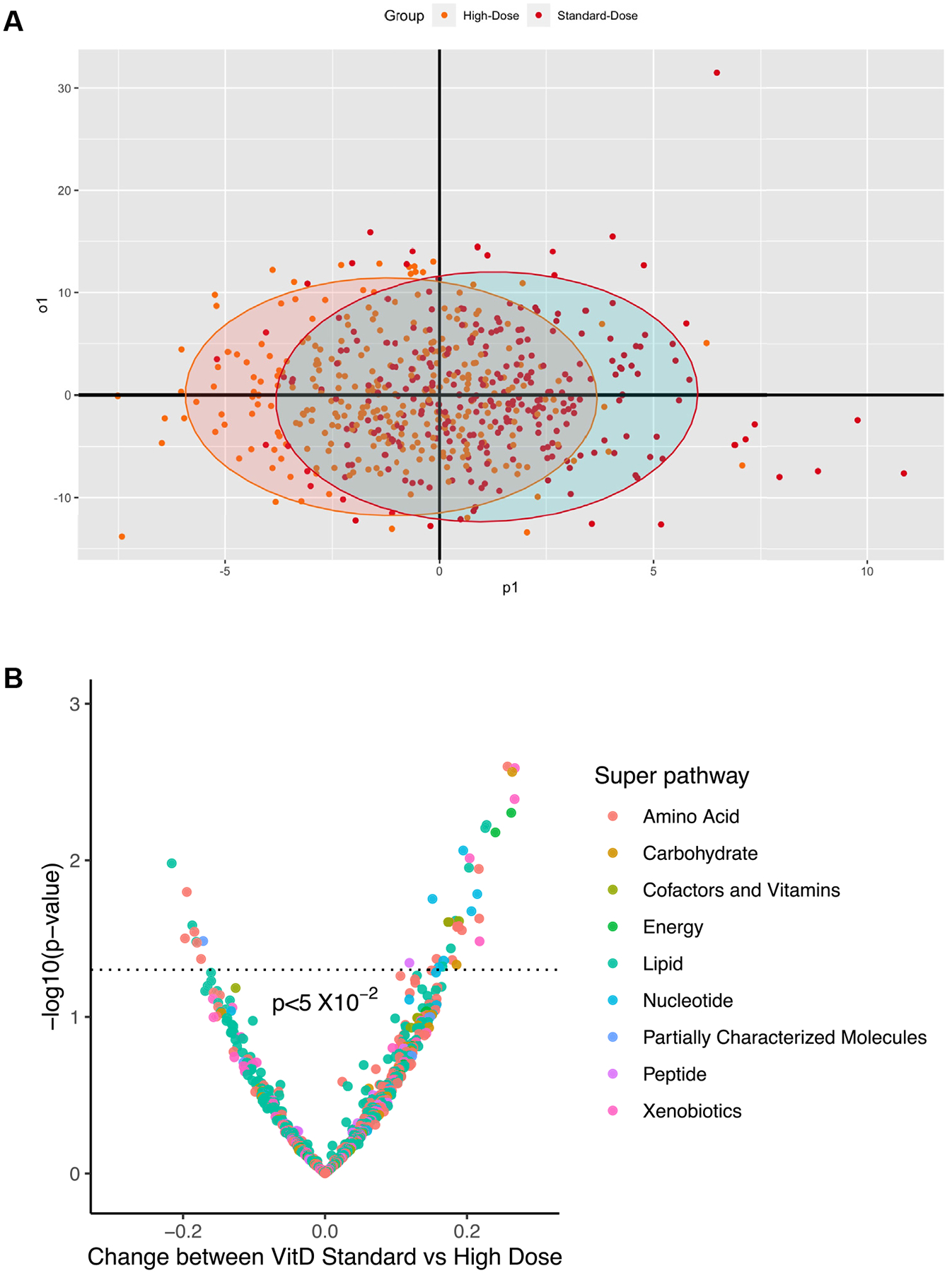
Maternal postpartum week 1 metabolome and vitamin D standard dose vs high-dose. **(A)** OPLS discriminant analysis score plot where separation between the 2 intervention groups is maximized along the predictive component (x-axis), while the orthogonal component accounts for intragroup variability (y-axis). Ellipses indicate the 95% confidence region for each intervention group. **(B)** Logistic regression models summarized in a volcano plot. Each dot represents a metabolite; different colors indicate metabolic super-pathways in which metabolites are involved. The x-axis indicates change in metabolite level (per SD) between the groups, while the y-axis indicates association strength in terms of −log10 of P value. No metabolite showed association at FDR 5% threshold. *VitD*, Vitamin D.

The metabolic profile (metabolites n = 753) of maternal blood at 1 week postpartum was analyzed in relation to high-dose versus standard-dose vitamin D_3_ supplementation using OPLS discriminant analysis with double 10-fold cross-validation and 1000 permutations, which showed no significant separation between the groups (permuted *P* >.05) (Fig 1, A). A subsequent univariate approach using confounder adjusted logistic regression models showed similar results with no significant findings at FDR 5% level (Fig 1, B).

### 25(OH)D level and maternal metabolic profile at 1 week postpartum in COPSAC_2010_

**FIG 2.**
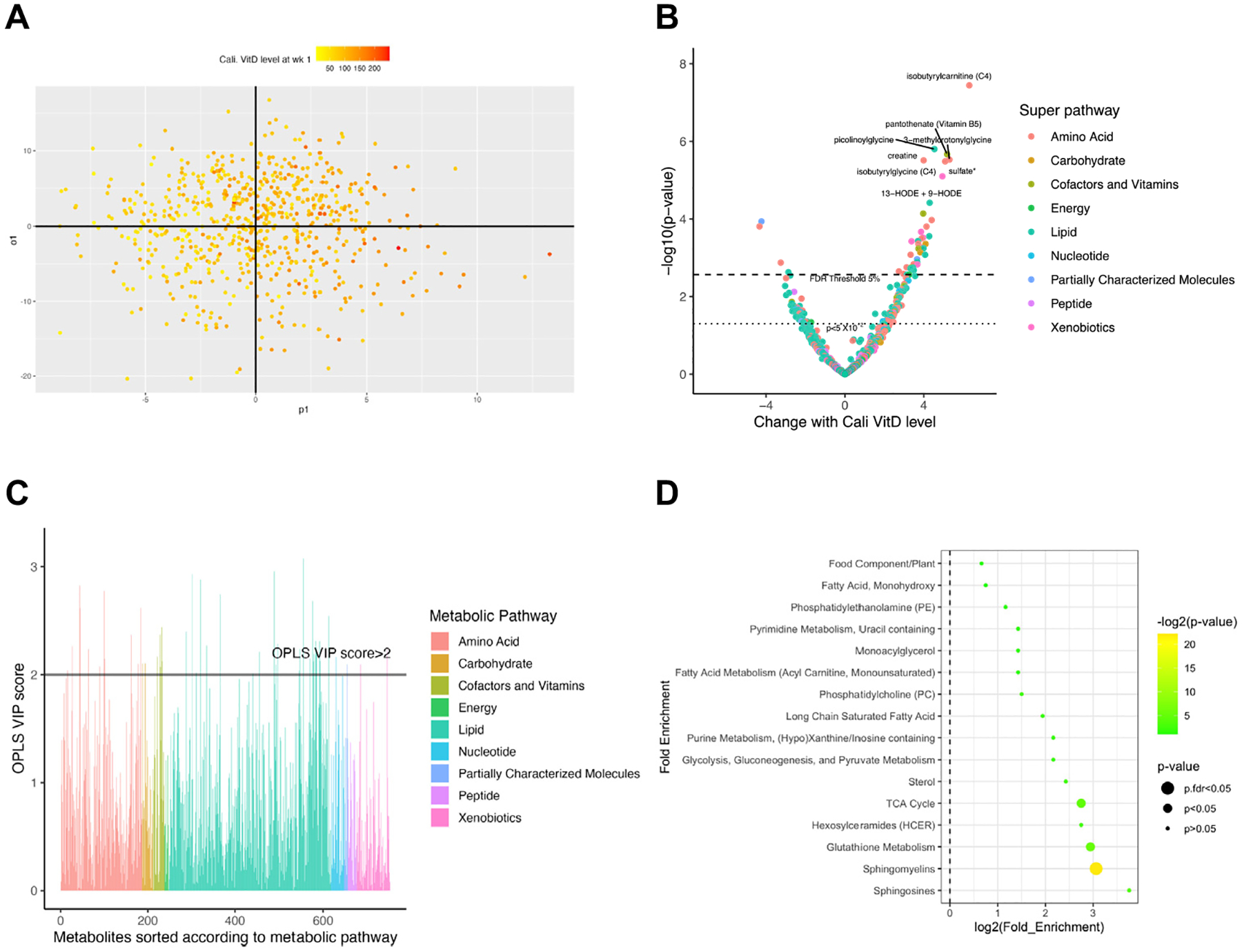
Maternal postpartum week 1 metabolome vs calibrated vitamin D level. **(A)** OPLS Y score plot where separation with calibrated vitamin D level (adjusted for vitamin D intervention, fish oil intervention, offspring sex, smoking during third trimester, and birth season) is maximized along the predictive component (x-axis), while the orthogonal component accounts for intragroup variability (y-axis). Color of each dot (each sample) represents levels of calibrated vitamin D level; intense red indicates relatively higher vitamin D level. Permutation test (n = 1000) showed no significance in the model (*P* > .05). **(B)** Linear regression models summarized into a volcano plot. Each dot represents a metabolite; different colors indicate metabolic super-pathways in which metabolites are involved. The x-axis indicates change in metabolite level (per SD) with calibrated vitamin D level (nmol^−1^), while the y-axis indicates association strength in terms of −log10 of *P* value. **(C)** Bar chart showing VIP score based on the predictive component from (*A*) for each metabolite. The x-axis represents each metabolite; different colors indicate the metabolic super-pathways in which metabolites are involved. The y-axis represents OPLS Y VIP score from its predictive component. *Horizontal line* indicates OPLS Y VIP score of 2 (metabolite number above this threshold, n = 46). **(D)** Pathway enrichment analysis based on metabolites with VIP score >2. The y-axis indicates the metabolic sub-pathway name, while the x-axis indicates logarithm of the enriched factor in each pathway. The bubble size and color indicate the *P* value. *Cali*, Calibrated; *TCA*, tricarboxylic acid; *VitD*, vitamin D.

First, an OPLS Y model was performed to investigate the calibrated 25(OH)D level versus the maternal metabolic profile at 1 week postpartum, which after cross-validation showed no significant separation (permuted *P* > .05) (Fig 2, A). However, the univariate approach using linear regression showed that 153 metabolites were significant at a nominal level (*P* < .05) and 42 metabolites were significant at FDR 5% level (Fig 2, B). Thus, the univariate analyses suggested that the associations of 25(OH)D may be limited to specific metabolic pathways.

Therefore, the 46 metabolites with VIP scores >2 were derived from the initial OPLS Y model (Fig 2, C) to build a PCA and a new OPLS Y model. A large degree of variance was explained in the PCA model by principal component 1 (PC1) (21.5%) and principal component 2 (PC2) (12.5%), where the metabolites with the greatest contribution to PC1 and PC2 were sphingomyelins (Fig E2 in the Online Repository at www.jacionline.org). Both PC1 and PC2 were positively associated with maternal calibrated 25(OH)D (PC1: β = 0.02, *P* = 1.93 × 10^−8^; PC2: β = 0.01, *P* × 7.86 3 10^−7^). Further, the new OPLS Y model showed a significant separation after cross-validation (*P* = .01), suggesting that the metabolic profile consisting of the 46 selected metabolites is affected by 25(OH)D exposure.

Finally, to identify metabolic sub-pathways influenced by 25(OH)D level, we conducted pathway enrichment analysis (Fig 2, D). From the 24 metabolic sub-pathways in which the 46 metabolites are involved, sphingomyelins and phosphatidylcholine (both lipid super-pathways) showed significant enrichment at nominal level. After multiple test corrections, only the sphingomyelin sub-pathway (12 of the 46 metabolites) showed significant enrichment at the FDR 5% threshold.

### 25(OH)D-related metabolites and offspring clinical end points in COPSAC_2010_

#### Primary end points.

**FIG 3.**
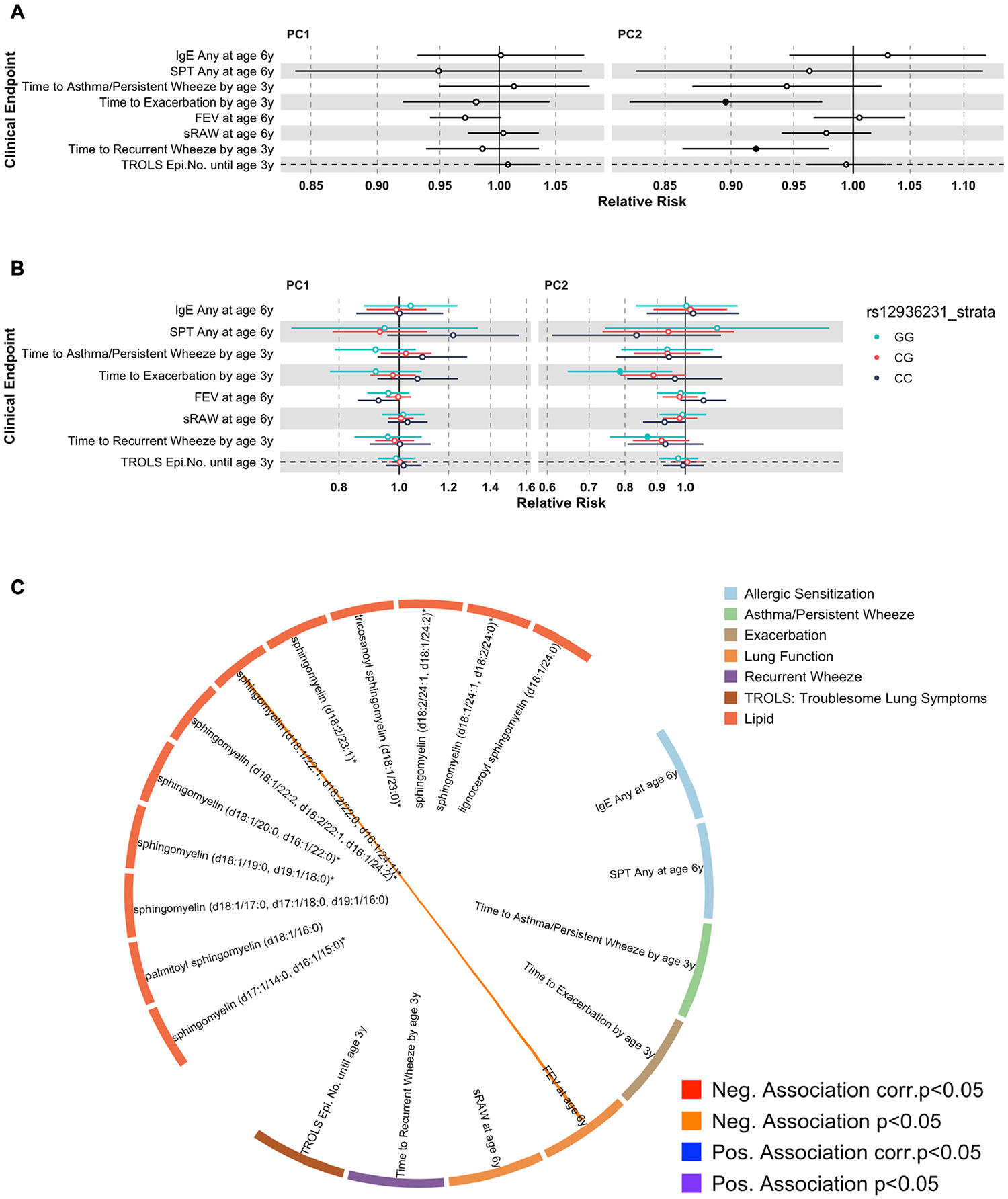
**(A** and **B)** Forest plots showing association between PC1 and PC2 scores from a maternal postpartum week 1 PCA model based on 46 metabolites that had a VIP score >2 in relation to calibrated vitamin D level and primary child clinical end points. Forest plot in (*A*) shows when not stratified by child 17q21 rs12936231 genotypes, while plot in (*B*) shows when stratified by child 17q21 rs12936231 genotypes. Regression models were used between PCA scores and clinical end points. The x-axis represents relative risk for each component score, while the y-axis represents each clinical end point. Each dot represents relative risk per unit of component score, while the error bar represents 95% CI of relative risk. Different colors represent rs12936231 genotypes. Cox regression models were used to test the association between the metabolites and persistent wheeze, asthma, and exacerbation end points. Linear regression models were used between the metabolites and lung function end points. Logistic regression models were used for allergic sensitization, while quasi-Poisson regression models were used for TROLS. PC1 and PC2 explained 21.5% and 12.5% of the variance, respectively. **(C)** Circos plot showing association between 12 sphingomyelins from maternal postpartum week 1 and child clinical end points. Twelve sphingomyelins were selected because the sphingomyelin pathway was the metabolic pathway most affected by vitamin D level, ie, significant enrichment, and 12 of them had VIP scores >2. *Orange line* represents negative association at nominal level (*P* < .05). *corr*., Corrected; *Epi.No*., number of episodes; *Neg*., negative; *Pos*., positive; *sRAW*, specific airway resistance.

The maternal metabolic profile based on the 46 25(OH(D)-related metabolites was investigated in relation to offspring clinical end points using PC1 and PC2 scores. PC2 showed significant associations with both recurrent wheeze (hazard ratio [HR] = 0.92 [95% CI 0.84–0.97], *P* = .02) and wheeze exacerbations (HR = 0.92 [95% CI 0.84–0.97], *P* = .02) (Fig 3, A; Table E1 in the Online Repository at www.jacionline.org). When stratified by child 17q21 rs12936231 genotype, associations between PC2 versus recurrent wheeze (HR = 0.87 [95% CI 0.76–0.99], *P* = .04) and wheeze exacerbation (HR = 0.79 [95% CI 0.64–0.95], *P* = .01) were significant only in children with low-risk GG genotype (Fig 3, B).

We subsequently tested the associations of the 12 sphingomyelins belonging to the pathway that showed significant enrichment with the primary clinical end points. None of these sphingomyelins were individually associated with recurrent wheeze, persistent wheeze/asthma, wheeze exacerbations, or number of TROLS episodes at ages 0 to 3. Sphingomyelin (d18:1/22:1, d18:2/20:0, d16:1/24:1) showed an inverse association with FEV_1_ at nominal level (relative risk = 0.89 [95% CI 0.80–0.98], *P* = .02), but did not pass FDR multiple test correction. There was no association between the sphingomyelins and the risk of allergic sensitization (Fig 3, C; Table E1).

#### Secondary end points.

PC1 and PC2 scores were not associated with any infection end points (Fig E3, A, in the Online Repository at www.jacionline.org). At an individual sphingomyelin level, increased palmitoyl-sphingomyelin (d18:1/16:0) was associated with reduced number of tonsillitis episodes (IRR = 0.78 [95% CI 0.64–0.95], *P* = .02), while increased tricosanoyl-sphingomyelin (d18:1/23:0) was associated with a reduced number of croup episodes (IRR = 0.79 [95% CI 0.62–1.00], *P* = .05) until age 3, but not at FDR 5% threshold (Fig E3, B, in the Online Repository at www.jacionline.org). Associations between levels of all of the 46 25(OH)D-related maternal metabolites versus all offspring clinical end points are shown in Fig E4, A (in the Online Repository at www.jacionline.org).

### Validation in VDAART

**FIG 4.**
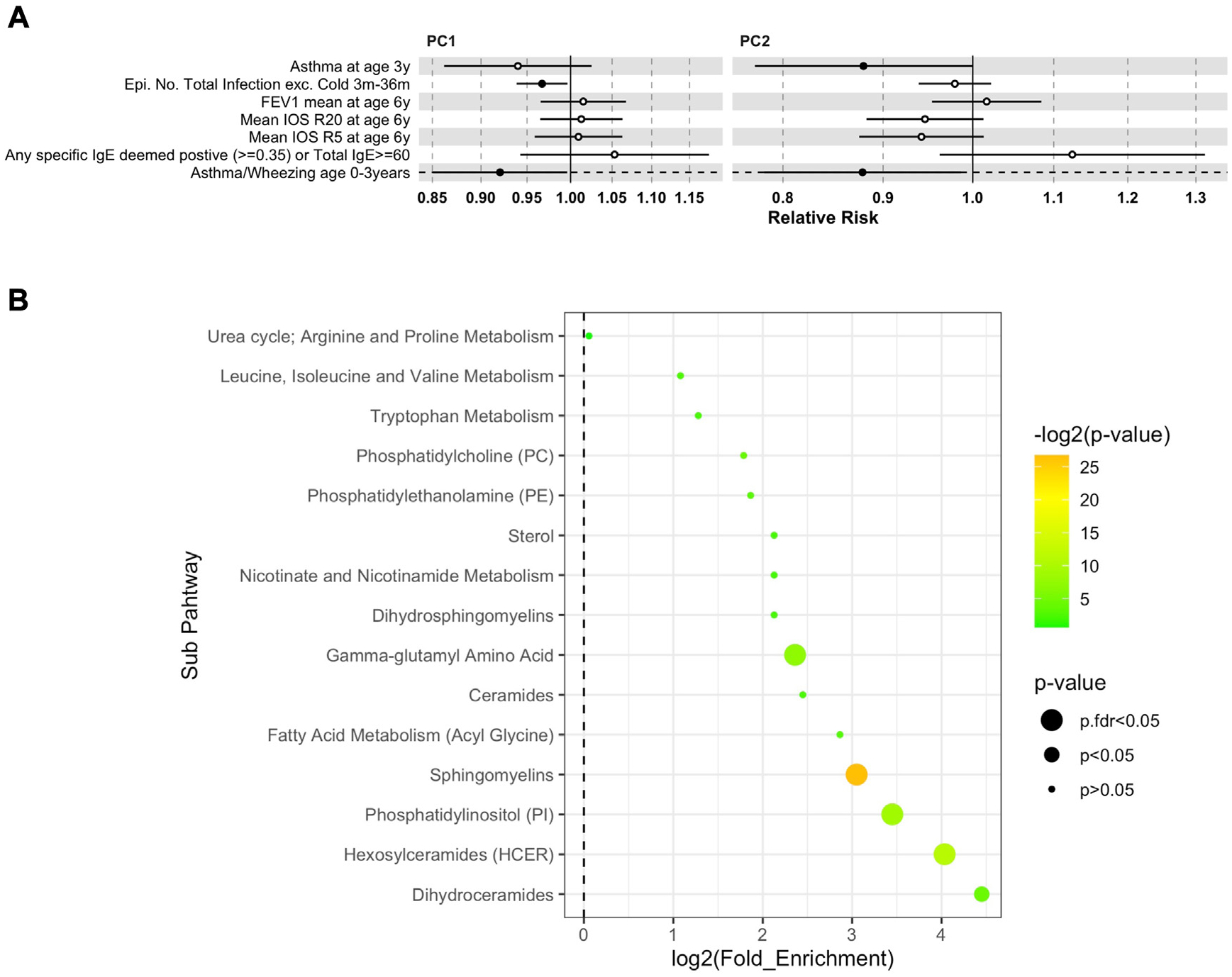
Findings in VDAART validation cohort. **(A)** Forest plot showing association between PC1 and PC2 scores from a maternal gestation weeks 32 to 38 PCA model based on 45 metabolites selected from COPSAC_2010_ findings. Each dot represents relative risk per unit of component score, while the error bar represents 95% CI of relative risk. Different colors represent types of clinical end points. Linear regression models were used between the metabolites and lung function end points. Logistic regression models were used for asthma wheezing by age 3y and asthma at age 3y, while quasi-Poisson regression models were used for number of infection episodes during age 3 months and 3 years. **(B)** Pathway enrichment analysis based on metabolites with VIP scores >2. The y-axis indicates the metabolic sub-pathway name, while the x-axis indicates logarithm of the enriched factor in each pathway. The bubble size and color indicate the *P* value. Sphingomyelin pathway was the enriched metabolic pathway showing strongest significance with vitamin D level at gestation weeks 32 to 38. *exc*, Excluding; *Epi.No*., number of episodes; *IOS R5*, impulse oscillometry system resistance at 5 Hz; *IOS R20*, impulse oscillometry system resistance at 20 Hz.

We used the VDAART cohort to validate the association between the maternal 1 week postpartum metabolomic 25(OH)D-related profile and clinical end points in COPSAC_2010_. Of the 46 selected metabolites in COPSAC_2010_, measurements on 45 metabolites were available at gestation weeks 32 to 38 in VDAART. PC1 and PC2 scores from a PCA model based on these 45 metabolites were derived. Both PC1 and PC2 were associated with asthma/wheeze at 0 to 3 years (PC1: odds ratio [OR] = 0.92 [95% CI 0.85–0.99], *P* = .04; PC2: OR = 0.88 [95% CI 0.78–0.99], *P* = .03), whereas PC2 was associated with asthma status at age 3 years (OR = 0.88 [95% CI 0.77–0.99], *P* = .049). PC1 was further associated with total number of infection episodes from age 3 months to 3 years (IRR = 0.97 [95% CI 0.94–0.99], *P* = .03) (Fig 4, A; Table E2 in the Online Repository at www.jacionline.org). No association was observed for lung function and sensitization end points. Associations between the 45 metabolites and offspring clinical end points in VDAART are shown in Fig E4, B (in the Online Repository at www.jacionline.org).

Additionally, we constructed an OPLS Y model between the whole metabolic profile and vitamin D level in VDAART, where 38 metabolites had a VIP score >2. Pathway enrichment analysis based on these 38 metabolites showed that sphingomyelin was the most significantly enriched metabolic pathway, which is consistent with the COPSAC_2010_ findings (Fig 4, B).

### 25(OH)D and its related metabolome in children at age 6 months

The associations between the maternal 1 week postpartum 25(OH)D-related metabolic profile and clinical end points in COPSAC_2010_ were reevaluated in a model adjusted for the effect of child age 6 months 25(OH)D level in addition to other confounder variables, PUFA intervention, maternal smoking, offspring breast-feeding days, sex, day care start age, and birth season. Fig E5, A (in the Online Repository at www.jacionline.org) shows only a subtle difference when compared with the previous multivariate models (Fig 3, A; Fig E3, A, in the Online Repository at www.jacionline.org), suggesting a minimal effect of 25(OH)D level in children on these associations.

Finally, we investigated whether the 25(OH)D-related metabolites found in the maternal metabolome were associated with child clinical end points using the child age 6 months metabolome as exposure. Of the 46 metabolites, 38 metabolite levels were available in the child metabolome, where PC1 and PC2 from a PCA based on these 38 metabolites showed no effects on recurrent wheeze or wheeze exacerbations (Fig E5, B, in the Online Repository at www.jacionline.org).

### Mechanistic study of vitamin D treatment in a human bronchial epithelial cell line

Given the observed metabolic profile enriched by sphingolipids after supplementation of high-dose 25(OH)D during pregnancy, we investigated whether this could be recapitulated in a cellular model. In human bronchial epithelial cells treated with various concentrations of 1*α*,25-vitamin D_3_ (0 nM, 0.1 nM, 1 nM, and 10 nM), we observed significant increases in levels of sphingosine-1-phosphate when cells were treated with high-dose 25(OH)D_3_ treatment models (0 nM vs 1 nM and 0 nM vs 10 nM) (*P* < .05 and *P* < .001, respectively) (Fig E6 in the Online Repository at www.jacionline.org).

## DISCUSSION

We performed an exploratory metabolomics study to investigate the biological pathways associated with the protective effects of high gestational vitamin D exposure on offspring respiratory health observed in the COPSAC_2010_ mother-child cohort. Increasing maternal 25(OH)D level at 1 week postpartum was characterized by a metabolic profile with enrichment of the sphingolipid pathway, more specifically sphingomyelins, which was further associated with a decreased risk of recurrent wheeze and wheeze exacerbations in the first 3 years of life even after adjusting for child age 6 months 25(OH)D level. The same 25(OH)D-related metabolic profile was also associated with reduced risk of asthma/wheeze in the independent VDAART mother-child cohort. Further, we found no significant associations between the 25(OH)D-related metabolic profile and recurrent wheeze and wheeze exacerbations using the child age 6 months metabolome in COPSAC_2010_, which underscores the importance of vitamin D exposure in the pregnancy period for promoting offspring respiratory health. Finally, we demonstrated a direct link between 25(OH)D_3_ treatment and cytoplasmic production of sphingolipids in human bronchial epithelial cells.

To our knowledge, only a few studies have previously investigated the association between 25(OH)D and the blood metabolome during pregnancy. One study showed an association between vitamin D and metabolites such as pyridoxate and N1-methyl-2-pyridone-5-carboxamide as well as 3-carboxy-4-methyl-5-propyl-2-furanpropanoate, which was also observed in our study. However, the study found no changes in sphingomyelins, potentially due to their limited metabolomics methodology including only 326 metabolites, while our study used a global profiling method detecting levels of 753 annotated metabolites including sphingomyelins. The association between vitamin D and sphingomyelins has been reported previously; for example, critically ill patients in the intensive care unit receiving a bolus of 540,000 IU of vitamin D showed changes in their blood metabolome dominated by sphingomyelins,^10^ and serum levels of sphingomyelins increased among overweight/obese Black after vitamin D supplementation in a dose-response fashion.^21^ Further, an observational study found lower levels of total sphingomyelins and dihydrosphingomyelins in serum samples of 25(OH)D-deficient middle-aged participants.^22^ These findings highlight a relationship between vitamin D and the sphingomyelin pathways.

While an extensive number of studies have been published on sphingolipid/sphingomyelin metabolism,^23^ the role of vitamin D in the sphingolipid pathways, specifically sphingomyelin metabolism, remains elusive. Cellular studies have demonstrated a critical role of the vitamin D receptor in sphingolipid metabolism,^24^ including some suggesting involvement of vitamin D metabolites in activation of the sphingomyelin pathway.^22,25^ In neurodegenerative disease, alterations of sphingolipids and 25(OH)D have been shown, and a structural analogue of sphingosine, which is a primary metabolite in sphingolipid metabolism, has been approved for multiple sclerosis treatment.^26^ Additionally, several animal model studies are under way in which sphingolipid metabolites and serine palmitoyltransferase agonists are exploited as a novel class of asthma therapeutics.^27–29^ All these studies highlight the importance of better understanding the role of sphingomyelin biology, especially in relation to asthma.

We found that the maternal 25(OH)D-related metabolic profile, which was enriched for sphingomyelins, was associated with a reduced risk of developing recurrent wheeze and wheeze exacerbations until age 3. This aligns with previous observational studies showing associations between sphingomyelins and asthma outcomes.^30,31^ Previously, we investigated the child blood metabolome at age 6 months in relation to asthma development later in life in the COPSAC_2010_ cohort, which showed an association between 21 sphingomyelins and risk of asthma development.^32^ Of these 21 sphingomyelins, 4 were also found in the current study. Additionally, 17q21 risk variants, which are the principal genetic risk factors for childhood asthma,^33^ are known to block serine palmitoyltransferase, which is a rate-limiting enzyme for *de novo* sphingolipid biosynthesis.^13,32,34^ We therefore carried out subsequent analyses where we stratified by child 17q21 rs12936231 genotype,^13,32^ which showed significant associations between the 25(OH)D-related metabolic profile and recurrent wheeze and wheeze exacerbations only among children with low-risk GG genotype, ie, only in children in whom the sphingomyelin pathway is not genetically blocked. This aligned with findings from our previous study^13^ that vitamin D exposure related to the maternal metabolome including sphingolipid pathways may have a greater downstream effect in children with the low-risk GG genotype and therefore possess a more protective effect on respiratory health. Finally, our mechanistic study showed a significant dose-dependent increase in cytoplasmic sphingolipid production when a human bronchial epithelial cell line was treated with 25(OH)D_3_, which supports a direct link between 25(OH)D_3_ treatment and the sphingolipid pathway in human airway epithelial cells.

The most comparable study to the present one is a previous analysis in VDAART investigating associations between the maternal blood metabolome at gestation weeks 32 to 38 and offspring asthma outcomes, which showed associations between 8 sphingomyelins and risk of offspring asthma or recurrent wheeze by age 3 years.^35^ The 8 sphingomyelins identified included palmitoyl-sphingomyelin (d18:1/16:0), lignoceroyl-sphingomyelin (d18:1/24:0), sphingomyelin (d18:2/24:1, d18:1/24:2), and sphingomyelin (d18:1/24:1, d18:2/24:0), which were all part of the 25(OH)D-related maternal metabolome in the current study. Importantly, we were able to validate our findings in VDAART, which emphasizes the role of the sphingomyelin pathway potentially explaining the link between prenatal 25(OH)D exposure and risk of offspring asthma/wheeze outcomes, but not allergy in early childhood. Further, in both COPSAC_2010_ and VDAART, we observed protective effects of the 25(OH)D-related maternal metabolites on the number of common childhood infections at a nominal significance level.

Sphingomyelins are known to play vital roles in cell signaling, vital in immunity and inflammation, and have been implicated in various disorders including atopic diseases,^36^ yet very little is known about their protective role in asthma development. Sphingomyelin can be hydrolyzed by acid sphingomyelinase (aSMase) to produce ceramide, while sphingomyelin synthase can convert ceramide back to sphingomyelin. Ceramide is a well-known proapoptotic molecule,^37^ while aSMase has been implicated in production of reactive oxygen species.^38^ Therefore, inhibition of aSMase activity and enhanced activity of sphingomyelin synthase leading to increased sphingomyelin levels could potentially be anti-inflammatory, leading to a reduced risk of childhood asthma development. Further targeting these pathways leading to higher sphingomyelin levels during the programming phases of pregnancy may have a long-lasting anti-inflammatory role in promoting offspring respiratory health.

Additionally, other sphingolipid pathways such as ceramides and sphingosines as well as tricarboxylic acid cycle–related metabolites were found to be associated with 25(OH)D level during pregnancy. Therefore, the effect of vitamin D exposure during pregnancy is not limited to sphingomyelins, as it affects multiple sphingolipid pathways as well as energy-related metabolic pathways, which have also been found to be implicated in asthma development.^13,32,39^

Overall, we employed metabolomics to uncover pathways related to the beneficial effects of high-dose vitamin D supplementation during pregnancy on risk of offspring TROLS, recurrent/persistent wheeze, asthma, and infections to support our previous findings,^6^ but only a subtle difference in the maternal metabolome was observed between the intervention groups. In contrast, we found a strong signal in the maternal metabolome using 25(OH)D levels measured 1 week postpartum, which may be due to lifestyle factors such as season and diet as well as genetics contributing to variation in 25(OH)D levels in addition to the intervention.^40^ The former is unlikely, as 25(OH)D level in maternal blood was adjusted for the season during which blood was collected. The latter, on the other hand, was not adjusted for due to lack of information on maternal diet between participant recruitment stage and 1 week postpartum.

This study has several strengths. First, to our knowledge, the study is the first to investigate underlying biochemical mechanisms potentially explaining the beneficial effect of high vitamin D exposure during pregnancy on asthma/wheeze-related outcomes in offspring occurring via maternal sphingomyelin metabolism, which was validated using an independent cohort. Second, the asthma/wheeze phenotypes were rigorously assessed using predefined algorithms, daily diary recordings, and objective assessments. Third, the availability of 2 cohorts for discovery and validation is a significant strength to rule out chance findings. Fourth, the inclusion of child 25(OH)D level and metabolic profiles at age 6 months underscored the importance of high maternal vitamin D exposure during pregnancy for promoting offspring respiratory health. Fifth, a cellular model showed a direct link between stimulation of a human bronchial epithelial cell line with increasing doses of 25(OH) and cytoplasmic production of sphingolipids.

### Conclusion

This exploratory metabolomics study shows that the beneficial effect of high gestational 25(OH)D levels on risk of asthma phenotypes in early life of offspring may occur via specific maternal metabolic alterations during pregnancy, including the sphingomyelin pathway. These results suggest the potential of targeting maternal sphingomyelin metabolism as a part of novel prenatal preventive strategies for reducing the incidence of childhood asthma.

## Supplementary Material

1

## Figures and Tables

**TABLE I.** Baseline characteristics of mothers and children in COPSAC_2010_ and VDAART

		Randomization*		
	All	Standard-dose vitamin D	High-dose vitamin D	Difference (*P* value)
**COPSAC**_**2010**_				
Mothers				
No.	672	276	286	
Age at birth (y), mean (SD)	32.3 (4.4)	32.0 (4.3)	32.5 (4.4)	.13^†^
Gestational age at birth, days, mean (SD)	279 (11)	279 (10)	77 (26)	.42^†^
Asthma, yes/no^‡^	179/492	73/202	78/208	.92^§^
Annual income, low/mid/high^ǁ^	63/164/102	25/68/37	26/62/45	.59^§^
Smoking, yes/no	22/649	13/262	8/278	.33^§^
Vitamin D level				
Pregnancy week 24 (nmol/L), mean (SD)	75.6 (25.2)	76.8 (25.5)	76.9 (25.4)	.95^†^
Postpartum week 1 (nmol/L), mean (SD)	88.2 (36.5)	73.3 (31.9)	106.6 (35.4)	< .0001^†^
Previous pregnancies, mean (SD)	1.19 (1.30)	1.15 (1.26)	1.30 (1.33)	.17^†^
Children				
No.	671	275	286	
Sex, female/male	327/344	136/139	138/148	81^§^
Birth season, winter/spring/summer/fall	207/175/142/153	100/51/53/71	104/51/61/70	.43^§^
Cesarean delivery, yes/no	145/527	57/218	65/221	.94^§^
Hospitalization at birth, yes/no	30/288	15/121	15/126	.98^§^
**VDAART**				
Mothers				
No.	779	236	223	
Age at birth, (y), mean (SD)	27.76 (5.53)	27.95 (5.56)	28.14 (5.46)	.71^†^
Asthma, yes/no^‡^	311/468	92/144	100/123	.20^§^
Site, Boston/San Diego/St Louis	226/269/284	70/84/82	69/72/82	.75^§^
Smoking, yes/no	18/754	43/181	44/173	.78^§^
Vitamin D level at gestation week 35 (ng/mL), mean (SD)	32.97 (14.56)	26.93 (10.96)	39.81 (14.98)	< .0001^†^
Children				
No.	459	236	223	
Sex, female/male	214/245	109/127	108/118	.73 ^§^
Race, Black/White/others	221/150/88	114/77/45	107/73/43	.99^§^
Birth season, winter/spring/summer/fall	176/169/191/243	56/62/52/66	56/51/53/63	.85^§^

* For COPSAC_2010_, the standard-dose group received 400 IU/day and the high-dose group received 2800 UI/day from pregnancy week 24 to postpartum week 1. For VDAART, the standard-dose group received 400 IU/day and the high-dose group received 4400 IU/day between 10 to 18 weeks of gestation and birth.

† test.

‡ History of physician-diagnosed asthma.

§ χ^2^ test.

ǁ Low (below €50 000), medium (€50 000- €110 000), high (above €110 000); €1 = US $1.07.

## Clinical implications:

Protective effects of increasing *in utero* vitamin D exposure were associated with a sphingomyelin-enriched metabolome, which was associated with better respiratory health outcomes.

## Abbreviations used

25(OH)D: 25-hydroxyvitamin D
aSMase: Acid sphingomyelinase
COPSAC_2010_: Copenhagen Prospective Studies on Asthma in Childhood_2010_
FDR: False discovery rate
HR: Hazard ratio
ICS: Inhaled corticosteroid
IRR: Incidence rate ratio
OPLS: Orthogonal partial least squares
OR: Odds ratio
PC1: Principal component 1
PC2: Principal component 2
PCA: Principal component analysis
PUFA: Polyunsaturated fatty acid
RCT: Randomized controlled trial
SPT: Skin prick test
TROLS: Troublesome lung symptoms
VDAART: Vitamin D Antenatal Asthma Reduction Trial
VIP: Variable influence on projection
