# Supplementary material for: Maternal vitamin D–related metabolome and offspring risk of asthma outcomes
Source: J Allergy Clin Immunol. Author manuscript; Available in PMC 2024 Jun 3. (PMC11147137; doi:10.1016/j.jaci.2023.06.030)
Supplement: 1 [file NIHMS1997564-supplement-1.pdf]

METHODS

Metabolomic data acquisition

Sample preparation. Metabolic profiling on EDTA plasma samples were prepared using the automated MicroLab STAR system from Hamilton Company (Reno, Nev). Recovery standards in methanol were added followed by vigorous shaking for 2 minutes (Glen Mills GenoGrinder 2000) followed by centrifugation. The resulting supernatant extract was divided into 5 fractions: 2 for analysis by 2 separate RP MS/MS methods with positive ion mode electrospray ionization (ESI), 1 for analysis by RP MS/MS with negative ion mode ESI, 1 for analysis by hydrophilic interaction chromatography (HILIC) MS/MS with negative ion mode ESI, and 1 reserved for backup. Samples were dried under nitrogen gas TurboVap (Zymark, Caliper, Hopkinton, Mass) overnight before analysis.

Metabolic profiling. Dried samples were reconstituted in starting mobile phase solvents for each method. Samples were analyzed using ACQUITY Ultra-Performance Liquid Chromatography (UPLC) (Waters, Milford, Mass) with Q Exactive Hybrid QuadrupoleOrbitrap mass spectrometer interfaced with heated electrospray ionization (HESI-II) source (ThermoFisher Scientific, Waltham, Mass). The sample extracts were reconstituted in solvents compatible to each of the 4 LC-MS methods used.

For RP in positive ESI mode optimized for more hydrophilic compounds, the extract was gradient eluted from a C18 column (Waters UPLC BEH C18-2.1 × 100 mm, 1.7 μm) using water and methanol, containing 0.05% perfluoropentanoic acid and 0.1% formic acid. For RP in positive ESI mode optimized for more hydrophobic compounds, the extract was gradient eluted from the same C18 column using methanol, acetonitrile, water, 0.05% perfluoropentanoic acid, and 0.01% formic acid and was operated at an overall higher organic content. For RP in negative ESI mode, a separate dedicated C18 column was used in which the basic extracts were gradient eluted from the column using methanol and water, both with 6.5 mM ammonium bicarbonate at pH 8. For HILIC in negative mode, a HILIC column (Waters UPLC BEH amide 2.1 × 150 mm, 1.7 μm) using a gradient consisting of water and acetonitrile with 10 mM ammonium formate, pH 10.8, was used. The MS/MS setting involved MS and data-dependent MSn scans using dynamic exclusion, scan range between 70 and 1000 m/z, at mass resolution 35,000.

Data extraction and compound identification. Raw data were extracted, peak identified, and quality control processed using Metabolon's hardware and software. These systems are built on a web-service platform using Microsoft .NET technologies, which run on high-performance application servers and fiber-channel storage arrays in clusters to provide active failover and load balancing. Compounds were identified by comparison with library entries of purified standards or recurrent unknown entities. Metabolon maintains a library based on authenticated standards that contains the retention time/index, mass to charge ratio (m/z), and chromatographic data (including MS/MS spectral data) on all molecules present in the library. Furthermore, biochemical identifications are based on 3 criteria: retention index within a narrow retention time/index window of the proposed identification, accurate mass match to the library ± 10 ppm, and the MS/MS forward and reverse scores between the experimental data and authentic standards. The MS/MS scores are based on a comparison of the ions present in the experimental spectrum with the ions present in the library spectrum. While there may be similarities between these molecules based on one of these factors, all 3 data points can be used to distinguish and differentiate biochemicals. More than 4500 commercially available purified standard compounds have been acquired and registered into LIMS for analysis on all platforms for determination of their analytical characteristics.

Data processing pipeline. A data normalization step was performed to correct variation resulting from instrument inter-day tuning differences by registering the medians to equal one and normalizing each data point proportionately. Further normalization was used to correct for analyzed sample volume variation. Median relative standard deviation (RSD) from quality control samples (pooled matrix samples) was determined to evaluate instrument variability (median RSD = 7%) and overall process variability (median RSD = 10%). We calculated missingness across all samples for each metabolite; metabolites with ≥30% missing values as well as unannotated metabolites were excluded. Missing values were imputed as half the minimum value across all samples for each metabolite. This resulted in a metabolic profile consisting of levels of 753 metabolites and subsequently used for further analyses.

Data analysis

After data quality control processing, data consisting of 753 metabolites were available for statistical analyses. Of more than 700 mothers enrolled into $COPSAC_{2010}$ cohort, 672 had plasma metabolic profiles available at both gestation week 24 and postpartum week 1 time points. Overall, data included levels of 753 annotated metabolites from 672 mothers at 2 points. Before statistical analyses, all metabolite levels were log-transformed to normalize the data distribution and *z*-scored to have mean of 0 and SD of 1.

Vitamin D exposure in maternal. First, we investigated change in blood metabolome with vitamin D (standard-dose vs high-dose intervention groups, calibrated vitamin D level) by employing 2 types of analysis models, multivariate and univariate. Fish oil intervention, maternal smoking during the third trimester, offspring sex, and birth season were calibrated to derive calibrated vitamin D level.

Multivariate data analysis was performed by using OPLS discriminant analysis for standard-dose versus high-dose intervention groups, and OPLS Y was used for blood vitamin D level. This allowed us to investigate how vitamin D influenced the blood metabolome as a whole. All models were subjected to double cross-validation (10-fold cross-validation, 1000 permutations) to test model validation. From each model, VIP scores toward the predictive component (which explains vitamin D) for each metabolite were derived.

Metabolites with VIP scores >2 were selected and underwent further OPLS models to test whether the metabolic profile consisting of these selected metabolites was associated with vitamin D. Further, pathway enrichment based on the hypergeometric test was carried out to identify metabolic pathways associated with vitamin D. All OPLS models were carried out using ropls R package.[E1] Afterward, a PCA model was built using the selected metabolites to identify the combined effect of key metabolites reflecting vitamin D.

Univariate data analysis was carried out using regression models and allowed us to investigate how vitamin D influenced levels of each blood metabolite. Logistic and linear regression models were employed for standard-dose versus high-dose intervention group comparison and calibrated vitamin D level, respectively. In logistic models, fish oil intervention, maternal smoking during the third trimester, offspring sex, and birth season were adjusted on the models. In linear models, the confounding variables were not input, as calibrated vitamin D level was already adjusted for these variables.

Maternal blood metabolome characterized by vitamin D versus offspring clinical end points. For the second part of this study, maternal metabolites with VIP scores >2 were investigated to test their association with offspring clinical end points. Cox regression survival analysis was performed to assess the association of metabolites with the risk of developing asthma, persistent wheeze, and exacerbation by age 3 years. Quasi-Poisson regression was applied to estimate the effect of key metabolites on relative incidence rate (IRR) of common infections and TROLS. Linear regression was used on lung function variables, and logistic regression was employed on sensitization (from SPT and IgE). All regression models were adjusted for child sex and pregnancy fish oil and vitamin D interventions.

All statistical analyses were performed in R Studio version 2021.09.2.

Vitamin D treatment of human bronchial epithelial cell lines

After starving cells overnight, we treated 16HBE wild-type control cells (ie, transfected with either empty vector or Cas-9 as controls) with 4 different concentrations of 1α,25-vitamin D_3 (Sigma-Aldrich) (0 nM, 0.1 nM, 1.0 nM, and 10.0 nM) for 10 hours in 12-well plates at 4×10^5 cells/well. Sphingosine-1-phosphate levels from cell lysates were measured by ELISA (MyBioSource). Experiments were repeated more than 10 times. Ordinary one-way ANOVA analysis followed by Dunnett comparisons was used to compare levels of sphingosine-1-phosphate between different concentrations.

REFERENCE

E1. Thévenot EA, Roux A, Xu Y, Ezan E, Junot C. Analysis of the Human Adult Urinary Metabolome Variations with Age, Body Mass Index, and Gender by Implementing a Comprehensive Workflow for Univariate and OPLS Statistical Analyses. J Proteome Res 2015;14:3322-35.

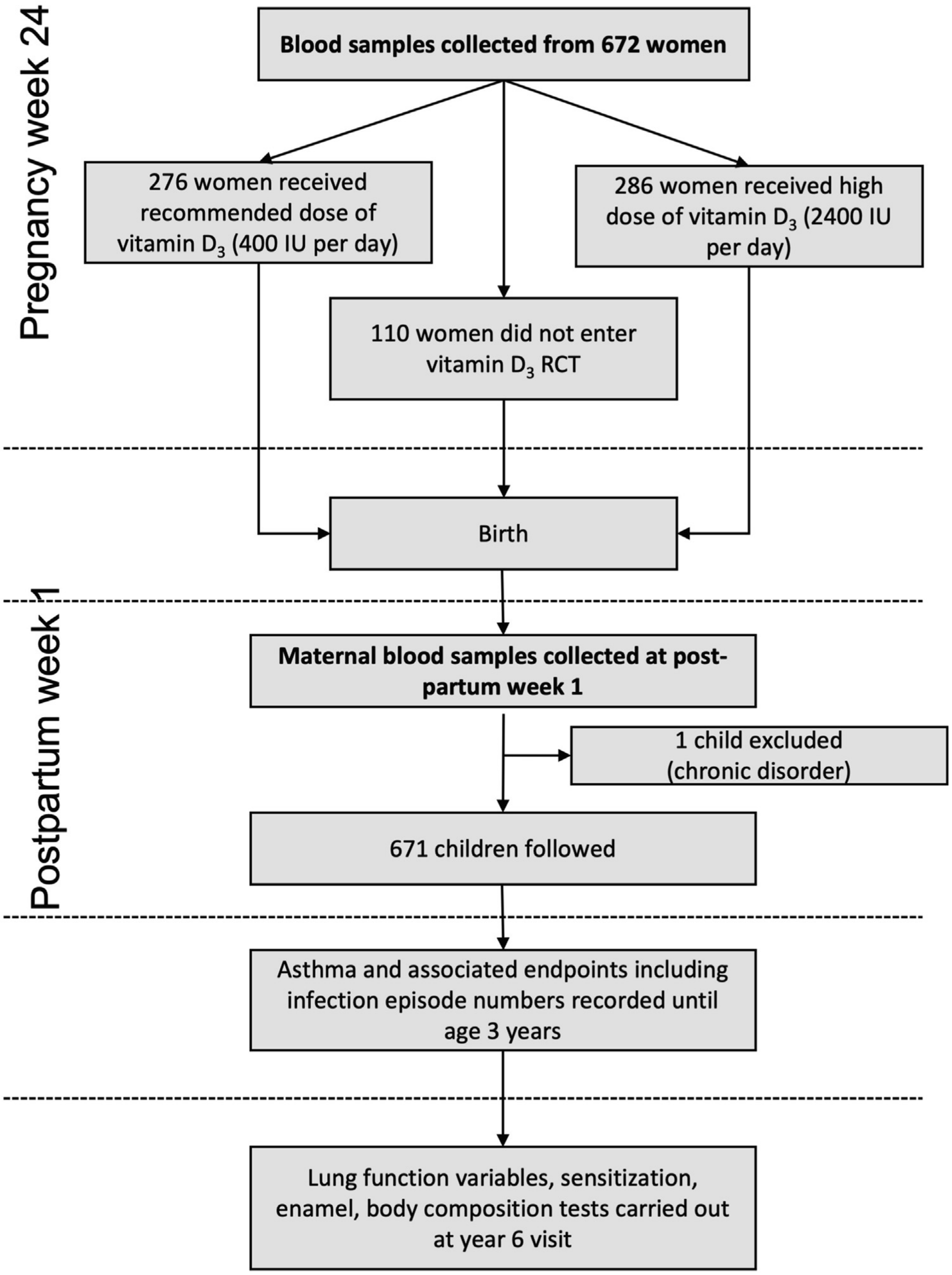

FIG E1. Flowchart of participants (mothers and children in $COPSAC_{2010}$) used in this study.

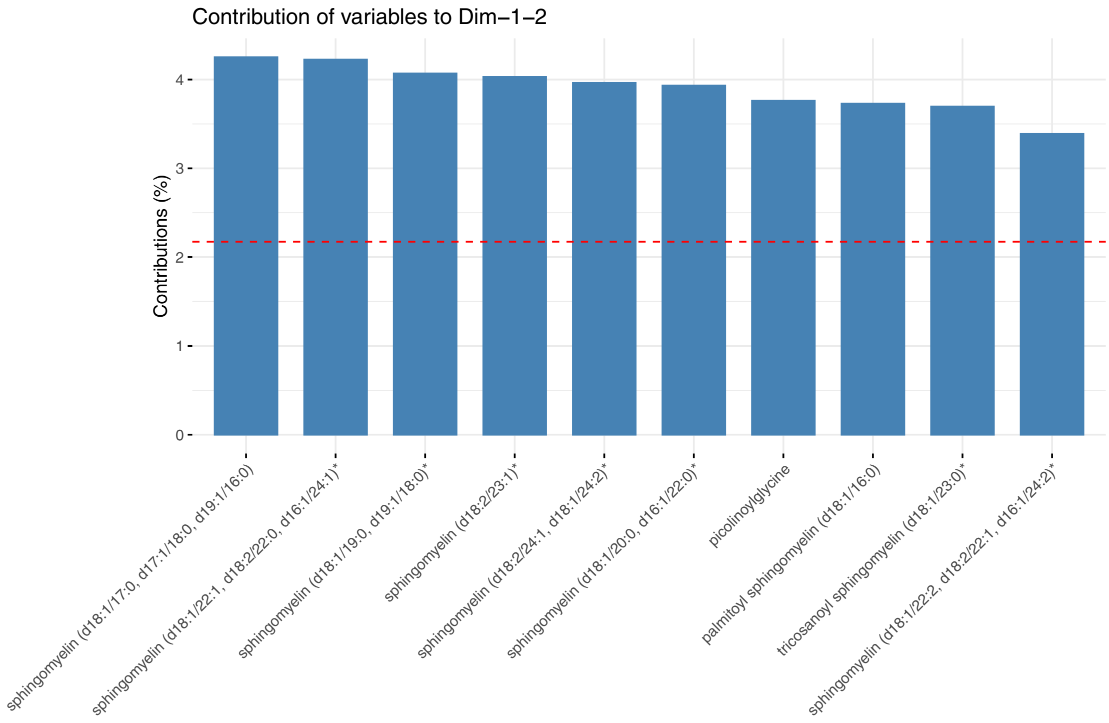

FIG E2. Bar chart showing top 10 metabolites contributing to the PCA model. The PCA model was based on levels of 46 metabolites derived from Fig 2. The x-axis represents metabolites, and the y-axis represents contribution (%) of metabolites to the model.

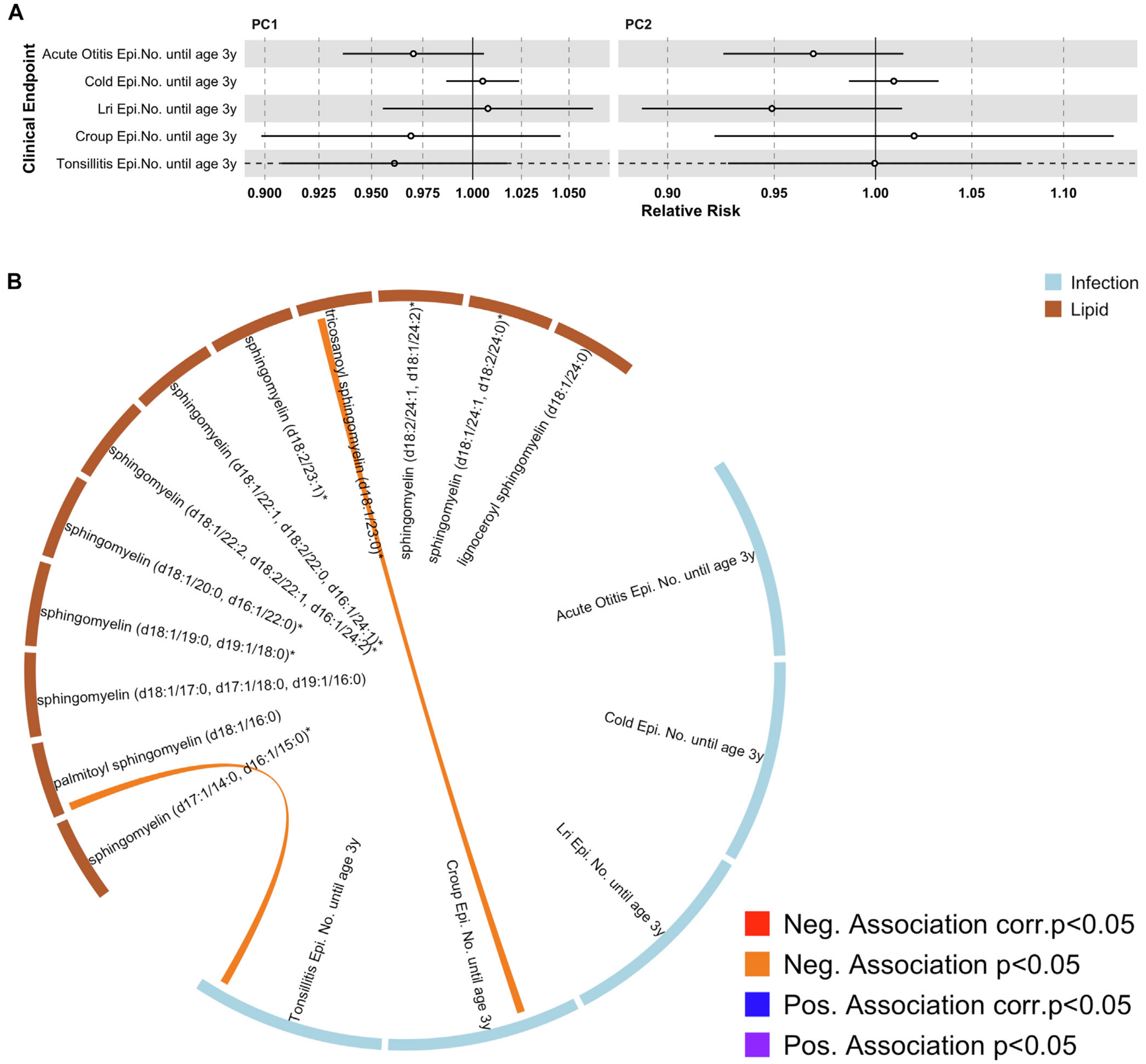

FIG E3. **(A)** Forest plots showing association between number of infection episodes and PC1 and PC2 scores from PCA model based on 46 metabolites. The x-axis represents relative risk for each component score, and the y-axis represents each clinical end point. Each dot represents relative risk per unit of component score, while the error bar represents 95% CI of relative risk. **(B)** Circos plot showing association between 12 sphingomyelins (from the panel of 46 metabolites) from maternal postpartum week 1 and child clinical end points. Twelve sphingomyelins were selected here because the sphingomyelin pathway was the metabolic pathway most affected by vitamin D level (Fig 2, *D*), and 12 of them had VIP scores >2 (according to Fig 2, *A* and *C*). Quasi-Poisson regression models were used for infection end points. *Orange line* (or *edge*) represents negative association at nominal significance level. *corr.,* Corrected; *Epi.No.,* number of episodes; *Lri,* lower respiratory infection; *Neg.,* negative; *Pos.,* positive.

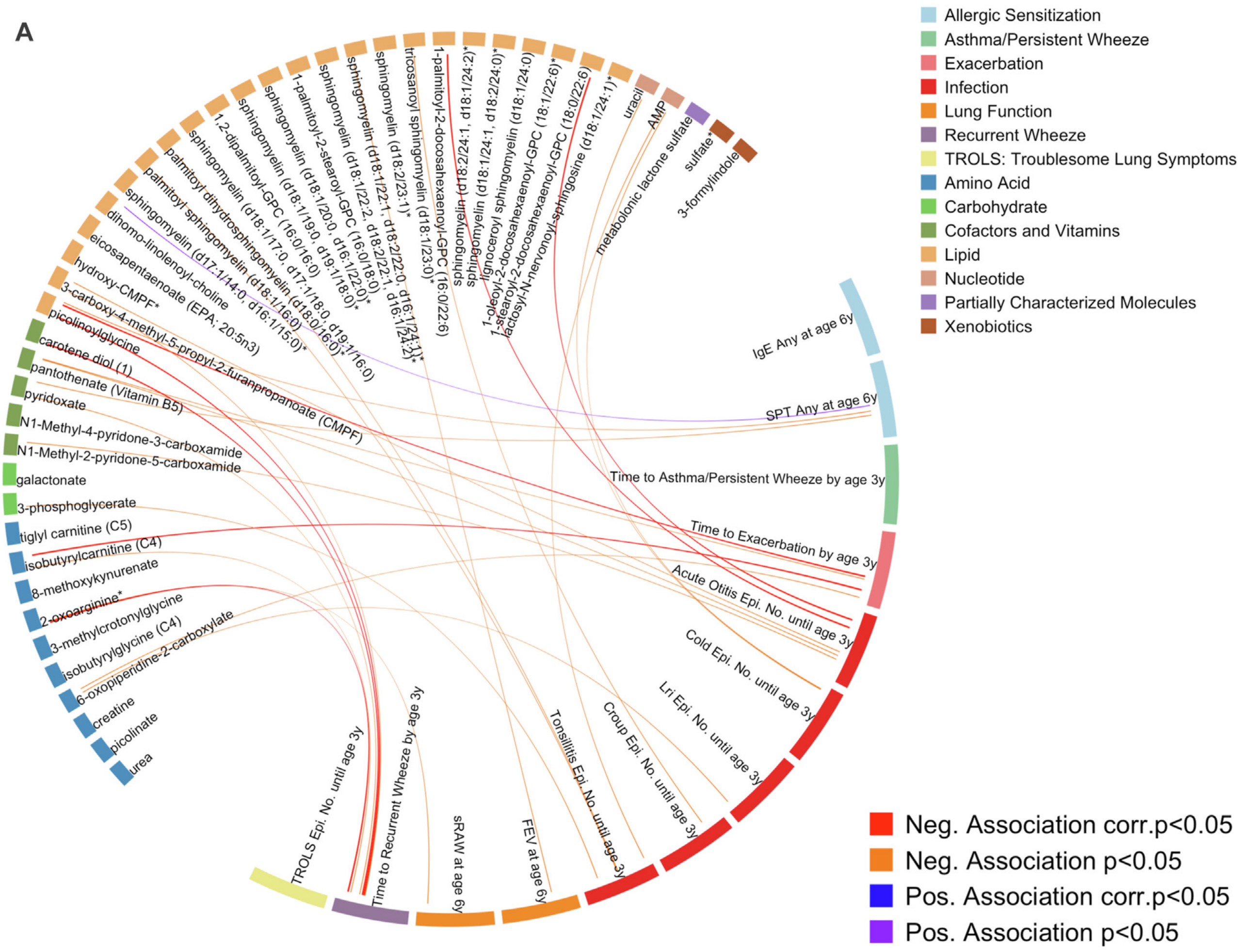

FIG E4. **(A)** Circos plot showing association between 46 metabolites from maternal postpartum week 1 and child clinical end points in $COPSAC_{2010}$. The 46 metabolites here were selected because they had VIP scores >2 (according to Fig 2, *A* and *C*). Cox regression models were used to test the association between the metabolites and persistent wheeze, asthma, and exacerbation end points. Linear regression models were used between the metabolites and lung function end points. Logistic regression models were used for allergic sensitization, while quasi-Poisson regression models were used for TROLS and infection. **(B)** Circos plot showing association between 46 metabolites from maternal postpartum week 1 and child clinical end points in VDAART. Here, 45 metabolites were selected (1 metabolite was not detected in the methodology). Multiple test correction was carried out based on the number of metabolic super-pathways involved by the 46 metabolites (n = 7). *corr,* Corrected; *Epi.No.,* number of episodes; *IOS R5,* impulse oscillometry system resistance at 5 Hz; *IOS R20,* impulse oscillometry system resistance at 20 Hz; *Lri,* lower respiratory infection; *Neg,* negative; *Pos,* positive; *sRAW,* specific airway resistance.

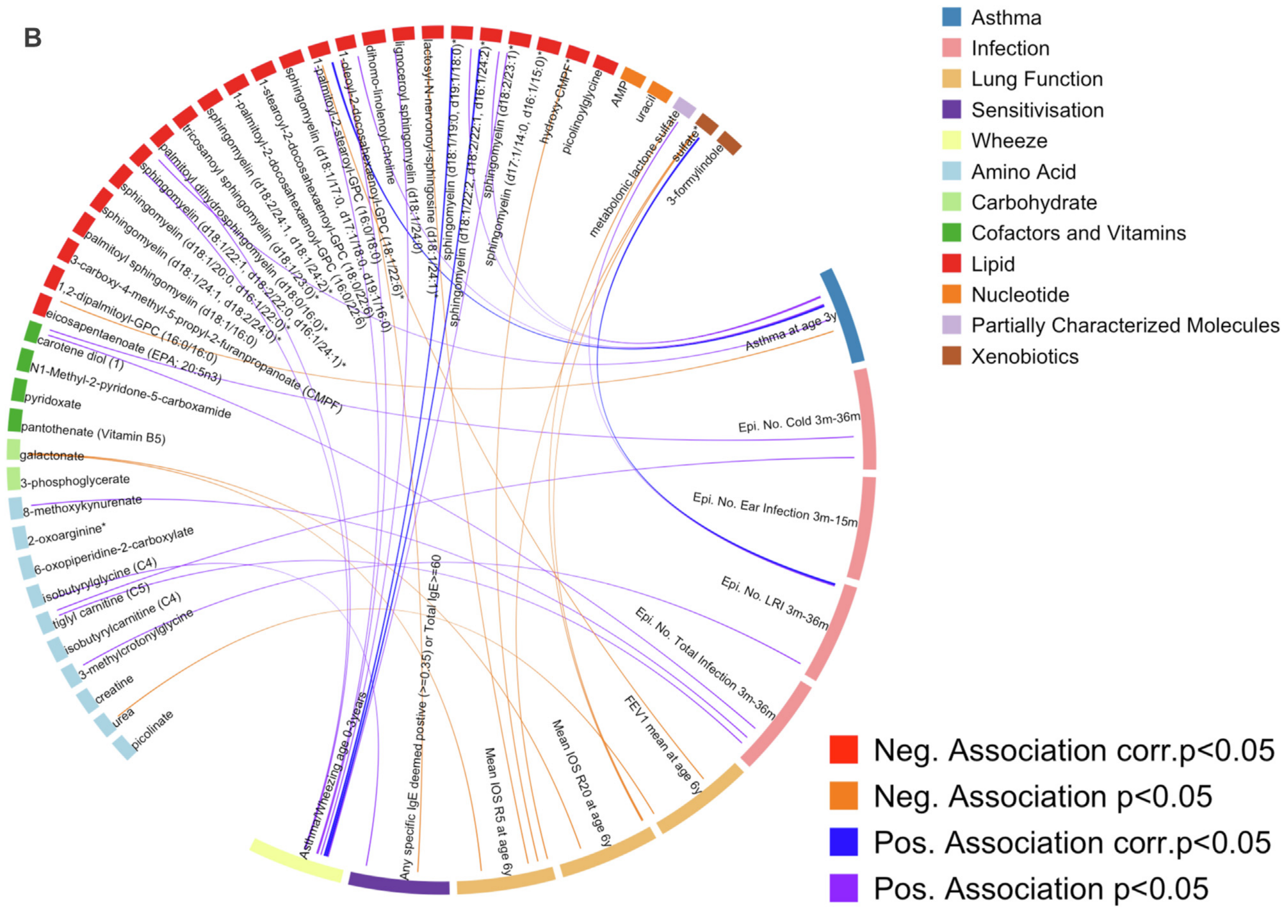

FIG E4. Continued.

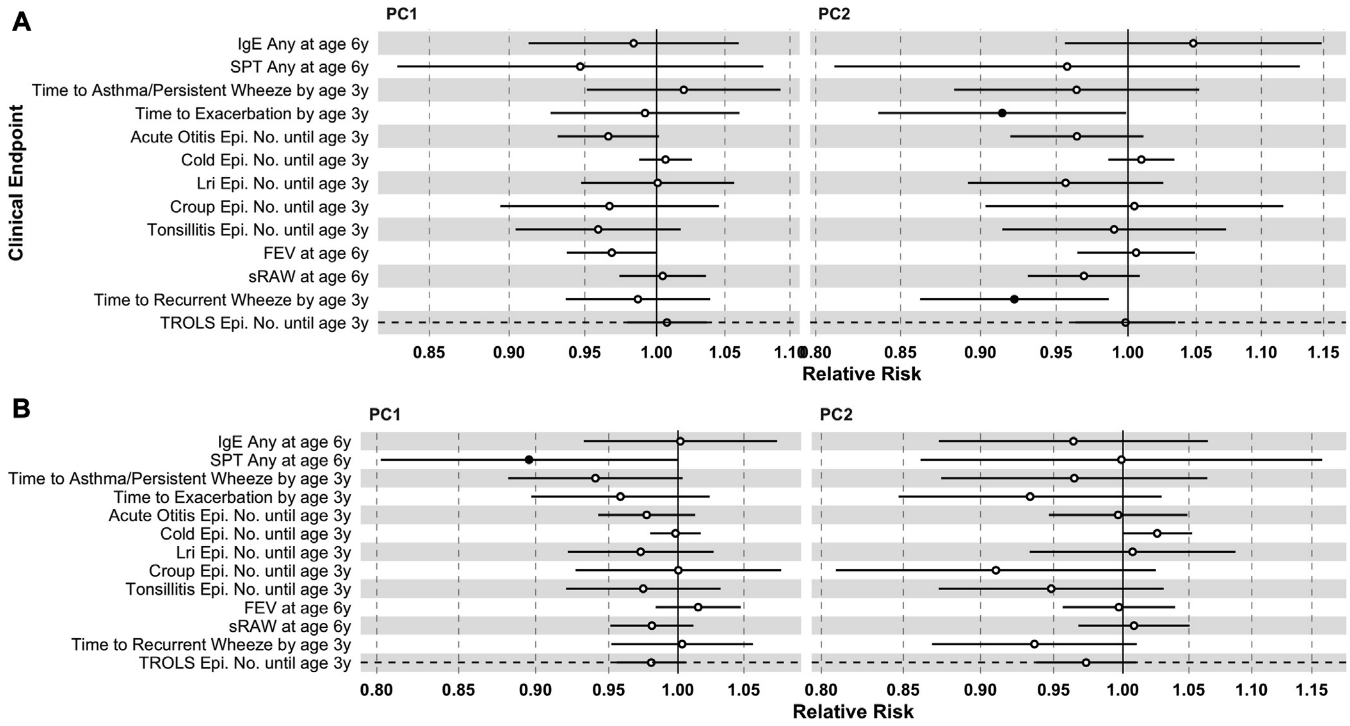

FIG E5. (A and B) Forest plots showing association between clinical end points and 25(OH)D-related metabolome in mothers at postpartum week 1 *(A)* and in children at age 6 months *(B)*. *(A)* The models are similar to those in Fig 3, *A*; here, the effect of 25(OH)D level in children at age 6 months was additionally adjusted for. *(B)* In the offspring metabolome, 38 of 46 metabolite levels were available. PC1 and PC2 scores were derived from PCA based on these 38 metabolites, and they were tested against clinical end points. The x-axis represents relative risk for each component score, and the y-axis represents each clinical end point. Each dot represents relative risk per unit of component score, while error bar represents 95% CI of relative risk. *Epi.No.,* Number of episodes; *Lri,* lower respiratory infection; *sRAW*, specific airway resistance.

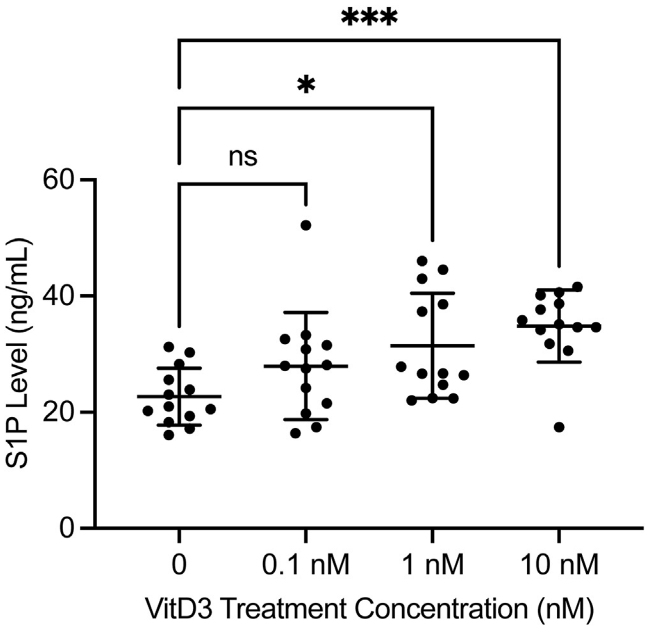

FIG E6. Box plot showing cytoplasmic levels of sphingosine-1-phosphate in human bronchial epithelial cells after vitamin D_3 treatment at 4 concentrations (0 nM, 0.1 nM, 1 nM, and 10 nM). Each dot represents each independent repeat. Ordinary one-way ANOVA analysis followed by Dunnett comparisons was used for the statistical analysis. ns, not significant, $P > .05$. $^{*}P < .05$, $^{***}P < .001$. *S1P,* Sphingosine-1-phosphate; *VitD3,* vitamin D_3.

TABLE E1. Association between maternal metabolome at postpartum week 1 and child primary clinical end points

	Wheeze age 0-3 years, HR [95% CI]		Exacerbation age 0-3 years, HR [95% CI]	TROLS episode number age 0-3 years, IRR [95% CI]	Lung function measurements at age 6 years, RR [95% CI]		Allergic sensitization, OR [95% CI]	
	Persistent	Recurrent			FEV_1	sRAW	IgE	SPT
**Multivariate**								
PC1	0.98 [0.92-1.05]	1.01 [0.95-1.02]	1.02 [0.96-1.09]	0.99 [0.97-1.02]	1.03 [0.99-1.06]	1.00 [0.97-1.03]	1.00 [0.93-1.07]	1.05 [0.93-1.19]
PC2	0.94 [0.87-1.02]	0.92 [0.84-0.97]*	0.89 [0.82-0.97]*	0.99 [0.96-1.03]	1.00 [0.97-1.05]	0.98 [0.94-1.02]	1.03 [0.95-1.12]	1.05 [0.83-1.12]
**Univariate**								
Lignoceroyl-sphingomyelin (d18:1/24:0)	1.12 [0.92-1.37]	1.02 [0.87-1.19]	1.06 [0.86-1.29]	1.03 [0.95-1.12]	0.93 [0.84-1.02]	1.03 [0.93-1.13]	0.92 [0.74-1.15]	1.08 [0.73-1.59]
Palmitoyl-sphingomyelin (d18:1/16:0)	1.03 [0.84-1.25]	0.99 [0.85-1.15]	0.96 [0.79-1.17]	1.01 [0.93-1.10]	0.95 [0.87-1.05]	1.02 [0.93-1.12]	0.99 [0.80-1.23]	0.86 [0.59-1.26]
Sphingomyelin (d17:1/14:0, d16:1/15:0)	1.12 [0.92-1.36]	1.00 [0.86-1.17]	0.96 [0.79-1.17]	1.03 [0.95-1.12]	0.92 [0.84-1.02]	1.00 [0.91-1.10]	0.97 [0.78-1.20]	1.00 [0.68-1.46]
Sphingomyelin (d18:1/17:0, d17:1/18:0, d19:1/16:0)	1.03 [0.84-1.25]	1.03 [0.88-1.2]	0.99 [0.81-1.21]	1.00 [0.92-1.08]	0.97 [0.88-1.07]	1.01 [0.91-1.11]	0.91 [0.73-1.14]	0.80 [0.54-1.18]
Sphingomyelin (d18:1/19:0, d19:1/18:0)	1.15 [0.94-1.4]	1.12 [0.96-1.31]	1.13 [0.92-1.39]	1.06 [0.97-1.15]	0.91 [0.83-1.01]	1.04 [0.94-1.14]	0.90 [0.72-1.12]	0.86 [0.58-1.27]
Sphingomyelin (d18:1/20:0, d16:1/22:0)	1.15 [0.93-1.4]	1.07 [0.91-1.25]	1.14 [0.93-1.4.]	1.07 [0.98-1.17]	0.92 [0.83-1.01]	1.03 [0.93-1.13]	1.01 [0.81-1.26]	0.81 [0.55-1.18]
Sphingomyelin (d18:1/22:1, d18:2/22:0, d16:1/24:1)	1.20 [0.97-1.48]	1.10 [0.93-1.29]	1.12 [0.9-1.38]	1.06 [0.97-1.16]	0.89 [0.8-0.98]*	1.05 [0.95-1.16]	0.96 [0.77-1.21]	1.12 [0.75-1.67]
Sphingomyelin (d18:1/22:2, d18:2/22:1, d16:1/24:2)	1.04 [0.86-1.25]	1.12 [0.96-1.30]	1.08 [0.89-1.3.]	1.04 [0.96-1.13]	0.92 [0.84-1.01]	1.04 [0.94-1.14]	0.98 [0.79-1.2]	0.94 [0.65-1.36]
Sphingomyelin (d18:1/24:1, d18:2/24:0)	0.96 [0.79-1.16]	0.95 [0.82-1.10]	0.93 [0.77-1.13]	1.00 [0.92-1.08]	0.93 [0.84-1.02]	1.04 [0.94-1.14]	0.98 [0.79-1.21]	0.71 [0.49-1.03]
Sphingomyelin (d18:2/23:1)	1.16 [0.95-1.41]	1.16 [0.99-1.36]	1.12 [0.91-1.37]	1.05 [0.97-1.14]	0.92 [0.83-1.01]	1.08 [0.98-1.18]	0.97 [0.78-1.2]	1.04 [0.7-1.53]
Sphingomyelin (d18:2/24:1, d18:1/24:2)	1.08 [0.89-1.31]	1.06 [0.91-1.24]	1.11 [0.9-1.35]	1.06 [0.97-1.15]	0.93 [0.84-1.02]	1.07 [0.98-1.18]	1.05 [0.85-1.31]	1.08 [0.74-1.58]
Tricosanoyl-sphingomyelin (d18:1/23:0)	1.09 [0.89-1.34]	0.99 [0.84-1.15]	1.01 [0.83-1.24]	1.01 [0.93-1.10]	0.92 [0.83-1.01]	1.01 [0.91-1.11]	0.84 [0.67-1.06]	0.97 [0.65-1.46]

All models are adjusted for sex of children and pregnancy fish oil and vitamin D interventions.

OR, Odds ratio; *RR,* relative risk; *sRAW,* specific airway resistance.

*Significant association at nominal level ($P < .05$).

TABLE E2. Association between maternal metabolome gestation week 35 and child clinical end points in VDAART

	Asthma at 3 years, OR [95% CI]	Asthma wheeze by 3 years, OR [95% CI]	Any specific IgE deemed positive (≥0.35) or total IgE ≥60, OR [95% CI]	FEV_1 at 6 years, RR [95% CI]	IOS R5 at 6 years, RR [95% CI]	IOS R10 at 6 years, RR [95% CI]	Total infection episode number excluding cold between 3 months and 3 years, IRR [95% CI]
PC1	0.94 [0.95-1.08]	0.92 [0.85-0.99]*	1.05 [0.95-1.18]	1.01 [0.97-1.07]	1.01 [0.96-1.06]	1.01 [0.96-1.7]	0.97 [0.94-0.99]*
PC2	0.88 [0.77-0.99]*	0.88 [0.78-0.99]*	1.12 [0.96-1.31]	1.02 [0.95-1.08]	0.95 [0.88-1.01]	0.94 [0.88-1.01]	0.98 [0.94-1.02]

All models are adjusted for sex of children, pregnancy vitamin D interventions.

IOS R5, Impulse oscillometry system resistance at 5 Hz; *IOS R20,* impulse oscillometry system resistance at 20 Hz; *RR,* relative risk.

*Significant association at nominal level ($P < .05$).
